# Patient outcomes following implantation with a trifocal toric IOL: twelve-month prospective multicentre study

**DOI:** 10.1038/s41433-018-0076-5

**Published:** 2018-09-06

**Authors:** M. Piovella, S. Colonval, A. Kapp, J. Reiter, F. Van Cauwenberge, J. Alfonso

**Affiliations:** 1 Centro di Microchirurgia Ambulatoriale, Monza, Italy; 2 CH De Jolimont-Lobbes Site Lobbes, Lobbes, Belgium; 3 Augenzentrum Michelfeld, Michelfeld, Germany; 4 Augen-Medizinisches Versorgungszentrum, Landshut, Germany; 5 CHU Sart-Tilman, Service d’Ophtalmologie Liège, Liège, Belgium; 6 Instituto Oftalmológico Fernández-Vega, Oviedo, España Spain

## Abstract

**Purpose:**

To evaluate clinical outcomes with a premium diffractive–refractive trifocal toric intraocular lens (IOL) over a 12-month period.

**Methods:**

Multicentre prospective clinical trial including 227 eyes of 114 patients undergoing cataract surgery with bilateral implantation of the AT LISA tri toric 939MP IOL (Carl Zeiss Meditec, Jena, Germany). One patient was implanted unilaterally. Outcome measures were: visual acuity, manifest refraction, reading performance, contrast sensitivity, defocus curve, patient satisfaction and subjective quality of vision. Alpins vector analysis was used to evaluate astigmatic changes.

**Results:**

12-month follow up results of binocular uncorrected distance, intermediate and near visual acuity were ≤0.3 logMAR in 99.0%, 98.10% and 91.40% of eyes, respectively. 79.7% of eyes had a cylinder value of ±0.50 D at 12 months post-surgery. Contrast sensitivity was in the normal range at 6 months post-surgery. The defocus curve exhibited a smooth transition between far and near foci. Vector analysis showed a mean magnitude of error of −0.16 ± 0.48 D. Mean binocular distance-corrected reading visual acuity was 0.15 ± 0.13 logRAD at 6 months postoperatively. 93.3%, 89.4% and 84.6% of patients expressed satisfaction (good or very good) with distance, intermediate and near vision, respectively, 12 months after surgery. Most (≥95%) patients felt that visual disturbances, including halos, glare, focusing difficulties and depth perception, caused little or no disturbance.

**Conclusions:**

The diffractive–refractive trifocal toric IOL, AT LISA tri toric 939MP, provides effective distance, intermediate and near visual acuity in eyes with corneal astigmatism. Patient satisfaction was high and 98.1% of patients expressed satisfaction with the IOL implanted.

## Introduction

Modern cataract and refractive surgery has enjoyed major advances in both surgical methods and in intraocular lens (IOL) development. It is now possible for cataract or presbyopic patients with corneal astigmatism to undergo microincison surgery with the implantation of an IOL with premium features. This means that postoperative complications are reduced and concommitantly, patient expectations have increased, with many patients undergoing surgery today expecting spectacle independence. Pre-existing corneal astigmatism is an important limiting factor in planning cataract surgery outcomes for a significant number of patients [1]. Treatment of astigmatism with toric IOL implantation provides effective visual restoration for these patients [2–6]. Toricity, combined with multifocality, seeks to provide complete visual restoration [7]. Premium diffractive–refractive trifocal toric IOLs are designed for micro-incison surgery and are an excellent option for presbyopia correction in eyes with significant amounts of corneal astigmatism [8–12]. The trifocal toric IOL AT LISA tri toric 939MP presents a trifocal anterior surface combined with toricity on both anterior and posterior surfaces and provides refractive correction at all distances [13]. To date, only 3-month outcomes with toric multifocal IOLs have been published [8–11] as well as one 12-month study limited to twenty patients [12]. The current study provides an in-depth analysis of 114 patients over a 12-month period of a premium diffractive–refractive trifocal toric IOL. Visual outcomes, manifest refraction, astigmatic changes, contrast sensitivity, photic phenomena and patient satisfaction were evaluated.

## Methods

### Patients

In this multicentre trial, with centres in Italy, Germany, Belgium, France and Spain, all patients (114) underwent uncomplicated phacoemulsification surgery. Patients had bilateral implantation, except one patient who had unilateral implantation, of the trifocal toric IOL AT LISA tri toric 939MP (Carl Zeiss Meditec, Jena, Germany). Inclusion criteria were patients aged 50 years or older, with bilateral cataractous eyes presenting bilateral regular corneal astigmatism, requiring surgical treatment and implantation of IOLs with a sphere power ranging between +0.0 and +28.0 D and cylinder power between +1.0 and +4.0 D. Cataract density had to be compatible with optical biometry evaluation. Exclusion criteria were monocular patients, previous ocular surgery, chronic or recurrent uveitis, acute ocular disease or external/internal infection, any kind of macular degeneration and impairment of retina, glaucoma or intraocular pressure (IOP) >21 mmHg under ocular hypertension treatment and any other at-risk pathology. All patients were adequately informed about the study and signed a consent form. The study adhered to the tenets of the Declaration of Helsinki and the ethics committee of each participating centre approved it. The trial is registered under the World Health Organization international clinical trials registry platform: NCT02770923.

### Examination protocol

Before surgery a complete ophthalmological examination was performed. Patients were evaluated postoperatively at 1 to 7 days and at 1 M, 3 M, 6 M and 12 M (M = month). One to seven days after surgery, the examination was performed for both eyes separately, including monocular subjective refraction, monocular uncorrected distance visual acuity (UDVA) and corrected distance visual acuity (CDVA), and slit lamp examination. At 1 M, 3 M, 6 M and 12 M postoperatively, monocular and binocular UDVA and CDVA, manifest refraction, binocular distance-corrected intermediate visual acuity (DCIVA) and uncorrected intermediate visual acuity (UIVA) at 80 cm and at preferred distance, binocular distance-corrected near visual acuity (DCNVA) and uncorrected near visual acuity UNVA at 40 cm, binocular DCNVA at preferred reading distance were performed. Corneal topography biomicroscopic examination with analysis of corneal status and IOL position (centration, tilt and axis position), were assessed subjectively by slit lamp examination.

The location and intensity of posterior capsule opacification (PCO) was evaluated using slit lamp examination under mydriasis. Loss in BCVA (yes/no) resulting in Nd:YAG capsulotomy (yes/no and time between cataract surgery and capsulotomy) was evaluated at M1, M3, M6 and M12.

Binocular reading performance with the Radner Reading Charts at 40 cm, binocular contrast sensitivity under photopic (80–160 cd/m^2^) and mesopic conditions (3 cd/m^2^) (Optec 6500 Vision Tester, Stereo Optical, Chicageo IL, USA), and measurement of the defocus curve measurements (from −4.0 to +1.0 D) at M3 and M12 were done.

Patients were asked to evaluate quality of vision at M3 and M12 and to describe their level of satisfaction with surgery and their level of spectacle independence using a subjective in-house questionnaire. Patient satisfaction was measured as very good, good, mediocre, bad or very bad. Patients answered questions at 3-months and 12-month postoperatively to determine their perception of halos and glare, in terms of frequency, severity and whether they were bothersome.

Subjective halo and glare score was analysed using Halo & Glare simulator computer software (Eyeland-Design network GmbH) at 1 and 6 months after surgery. The patients assessed their night visual perception by scaling halo and glare symptoms, moving an arrow that is linked to the image perceived in terms of size and intensity, from 0 to 100, where 0 means no halo or glare and 100 corresponds to severe halo or glare.

### Intraocular lens

The trifocal toric IOL evaluated (AT LISA tri toric 939MP, Carl Zeiss Meditec AG, Germany) is a 4-haptic design IOL, with an overall length of 11.0 mm and a 6.0-mm diameter optic, made of foldable hydrophilic acrylic material. AT LISA tri toric 939MP has a trifocal anterior surface with an add of 3.33 D for near and of 1.66 D for intermediate distance, both calculated at the IOL plane. It has an equiconvex bitoric optic and axis markers on the posterior side of the lens to guide its appropriate positioning within the capsular bag. The toric surface is distributed over the anterior and posterior surfaces and thus provides a larger usable biconvex optic on the anterior surface and, in addition, produces better modular transfer functions (MTF) for higher cylinders. AT LISA tri toric 939MP is an aspheric (aberration correcting −0.18 µm) IOL, has a square edge design and a 360° anti-posterior capsular opacification (PCO) ring on the optic. The IOL model used in this study is the pre-loaded version (MP) with a spherical power from −10.0 to +28.0 D, in 0.5 D increments, and cylinder power from +1.0 to +4.0 D, in 0.5 D increments.

### Data and statistical evaluation

The analyses were computed with SAS version 9.3 (SAS Institute Inc, Cary, NC, USA). The Kolmogorov-Smirnov test was used to check the normality of the data distributions. When parametric analysis was possible, the Student *t* test for paired data was performed for all parameter comparisons between preoperative and postoperative examinations as well as between consecutive postoperative visits. Otherwise, the Wilcoxon Rank Sum test was applied to assess the significance of differences between examinations. All statistical analyses were performed at the 5% global significance level, using two sided tests. In all cases, the same level of significance (*p* < 0.05) was considered.

Bilateral regular corneal astigmatism was confirmed by topography measurement. Values of the corneal radii (IOL Master Carl Zeiss Meditec, Germany) were taken at preoperative and at follow-up visits. The analysis of astigmatic changes was calculated using Excel and then analysed with the SAS software version 9.3 (SAS Institute Inc., Cary, NC, USA). allowing the analysis of the effectiveness of the astigmatic correction according to the Alpins method [14, 15]. The following vectors and parameters were calculated: targeted induced astigmatism (TIA), surgically induced astigmatism (SIA), and difference vector (DV), and magnitude of error (ME). SIA was assessed by comparing preoperative keratometer values to postoperative keratometer values, obtained using an IOL Master, at all follow up times, M1, M3, M6 and M12.

## Results

**Fig. 1:**
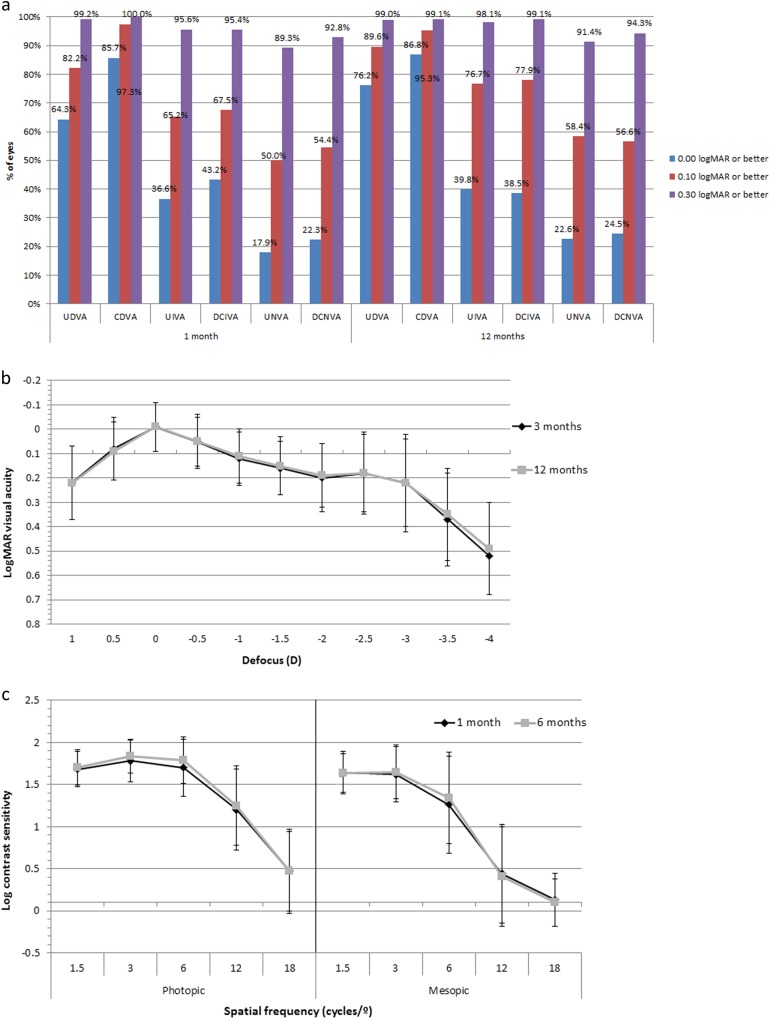
**a** Distribution of uncorrected distance (UDVA), intermediate (UIVA) and near visual acuity (UNVA) at 1 and 12 months after surgery in the analysed sample. **b** Mean defocus curve at 3 (black line) and 12 months (grey line) after surgery in the analysed sample. **c** Mean postoperative contrast sensitivity function measured under photopic (left) and mesopic (right) conditions at 1 month (blue line) and 12 months after surgery (red line) in the analysed sample

The study involved 227 eyes of 114 patients with a mean age of 63.7 ± 8.7 years (Fig. 1 supplemental). 36.8% were male and 63.2% of patients were female. Mean preoperative axial length (AL) and anterior chamber depth (ACD) were 23.53 mm (Standard deviation, SD: ±1.35) and 3.17 mm (±0.36), respectively. Mean preoperative photopic and mesopic pupil diameters were 3.31 mm (±0.85) and 4.79 mm (±0.88), respectively. The mean value of the spherical IOL power implanted was 19.79 D (±4.21) with a range of 5.0 D to 30.0 D. Cylinder IOL power ranged between 1.0 and 4.0 D with a mean value of 1.89 D (±0.83).

### Visual acuity and refractive outcomes

Table 1 summarizes the preoperative and postoperative visual and refractive data during the different time points of the 12-month follow-up. Stable values in monocular UDVA were observed at the different follow-up times post-surgery. There was a significant improvement in binocular DCIVA at 1 month post-surgery and the improvement was stable up to 12 months (*p* < 0.001). At 12 months post-surgery, 76.2% of eyes had fully restored (20/20 Snellen, 0.0 logMAR or better) UDVA, 39.8% has fully restored UIVA while 22.6% had fully restored UNVA (Fig. 1a).

**Table 1:** Summary of the visual and refractive outcomes in the sample evaluated

Mean (SD)	Preoperative	1–7 days	1 month	3 months	6 months	12 months	*P*-value
Monocular LogMAR UDVA	–	0.16 (0.17)	0.11 (0.14)	0.10 (0.14)	0.08 (0.13)	0.10 (0.14)	<0.001 (1–12 months)
Monocular LogMAR CDVA	0.24 (0.21)	0.07 (0.12)	0.02 (0.09)	0.02 (0.09)	0.01 (0.10)	0.02 (0.11)	<0.001 (preop-12 months)
							<0.001 (1–12 months)
Binocular LogMAR UDVA	–	–	0.04 (0.11)	0.03 (0.12)	0.02 (0.11)	0.02 (0.10)	0.159 (1–12 months)
Binocular LogMAR CDVA	–	–	−0.01 (0.08)	−0.02 (0.08)	−0.03 (0.09)	−0.02 (0.09)	0.004 (1–12 months)
Binocular LogMAR UNVA (40cm)	–	–	0.18 (0.14)	0.17 (0.14)	0.17 (0.14)	0.16 (0.14)	0.923 (1–12 months)
Binocular LogMAR DCNVA (40cm)	–	–	0.16 (0.13)	0.15 (0.13)	0.14 (0.13)	0.15 (0.13)	0.147 (1–12 months)
Binocular LogMAR DCNVA	–	–	0.15 (0.13)	0.14 (0.13)	0.13 (0.12)	0.13 (0.13)	0.933 (1–12 months)
(preferred distance)			(37.59±6.88)	(37.35±5.23)	(37.40±5.03)	(37.69±6.52)	
Binocular LogMAR UIVA (80cm)	–	–	0.09 (0.18)	0.08 (0.16)	0.08 (0.15)	0.06 (0.16)	0.052 (1–12 months)
Binocular LogMAR DCIVA (80cm)	0.15 (0.20)	–	0.06 (0.20)	0.04 (0.16)	0.05 (0.16)	0.04 (0.16)	<0.001 (preop-12 months)
							0.561 (1–12 months)
Binocular LogMAR UIVA	–	–	0.09 (0.17)	0.08 (0.16)	0.07 (0.14)	0.07 (0.15)	0.301 (1–12 months)
(preferred distance)			(71.75±10.42)	(69.90±9.76)	(69.49±7.92)	(69.88±7.57)	
Binocular LogMAR DCIVA	–	–	0.08 (0.18)	0.07 (0.16)	0.07 (0.15)	0.06 (0.15)	0.365 (1–12 months)
(preferred distance)			(69.04±10.79)	(69.05±9.81)	(68.93±7.93)	(69.53±7.52)	
Sphere (D)	0.22 (3.27)	−0.10 (0.52)	−0.10 (0.52)	0.03 (0.56)	0.03 (0.53)	0.01 (0.55)	0.065 (preop-12 months)
							0.07 (1–12 months)
Cylinder (D)	−1.19 (0.94)	−0.38 (0.42)	−0.32 (0.33)	−0.36 (0.35)	−0.38 (0.34)	−0.36 (0.34)	<0.001 (preop-12 months)
							0.143 (1–12 months)
SE (D)	−0.37 (3.35)	−0.29 (0.50)	−0.26 (0.48)	−0.15 (0.52)	−0.17 (0.48)	−0.18 (0.52)	0.688 (preop-12 months)
							0.003 (1–12 months)

*SD* standard deviation, *D* diopters, *UDVA* uncorrected distance visual acuity, *CDVA* corrected distance visual acuity, *DCNVA* distance-corrected near visual acuity, *DCIVA* distance-corrected intermediate visual acuity, *UIVA* uncorrected intermediate visual acuity, *SE* spherical equivalent

### Manifest refraction

A statistically significant reduction in manifest cylinder was found at 1 day after surgery (*p* < 0.001), with an additional significant reduction at 1 month postoperatively (*p* = 0.036). Table 1 gives a summary of the data over the 12-month follow-up and shows that the cylinder remained stable (*p* = 0.143). 82.2 and 79.7% of eyes had a cylinder value of ±0.50 D at 1 and 12 months post-surgery, and 95.2 and 97.8% of eyes had a cylinder value of ±1.00 D, at 1 and 12 months post-surgery, respectively.

### Defocus curves

Figure 1b shows the mean binocular defocus curves at 3 and 12 months post-surgery. The defocus curve of the trifocal AT LISA tri toric IOL demonstrates a smooth transition between the far and the near focus. The third focal point does not exhibit a “jump” in the visual acuity curve; there is a smooth transition phase from the far to the near focal point. Visual acuities better than 0.2 logMAR were observed for defocus levels greater than +1.00 D and less than −2.50 D demonstrating good intermediate vision. Near vision at 40 cm corresponds to the area of the curve at −2.5 D and at 3-months and 12-months the visual acuity values are >0.2 logMAR. Visual acuity values dropped to 0.5 logMAR when the defocus level was −4.00 D. The 3-month curve and the 12-month curve showed the same smooth transition between far and near foci and only deviate from each other at defocus levels less than −3.00 D. At −4.00 D the visual acuity equivalent is 25 cm. Defocus curves are not fully representative of reading visual acuity as the effects of convergence and pupillary constriction are not taken into consideration.

### Contrast sensitivity outcomes

Figure 1c demonstrates the mean contrast sensitivity function under photopic and mesopic conditions at 1 and 6 months post-surgery. Eyes were adapted for 10 min in the dark before mesopic measurements. Under both conditions, contrast sensitivity was similar at each follow-up (*p* > 0.05). The photopic contrast sensitivity curves were within the normal range with the exception of the values measured at high frequency (12 and/or 18 cpd). The reduction of contrast sensitivity at high spatial frequencies was also reported in a study with the non-toric version of the trifocal IOL [16].

### Photic phenomena

**Fig. 2:**
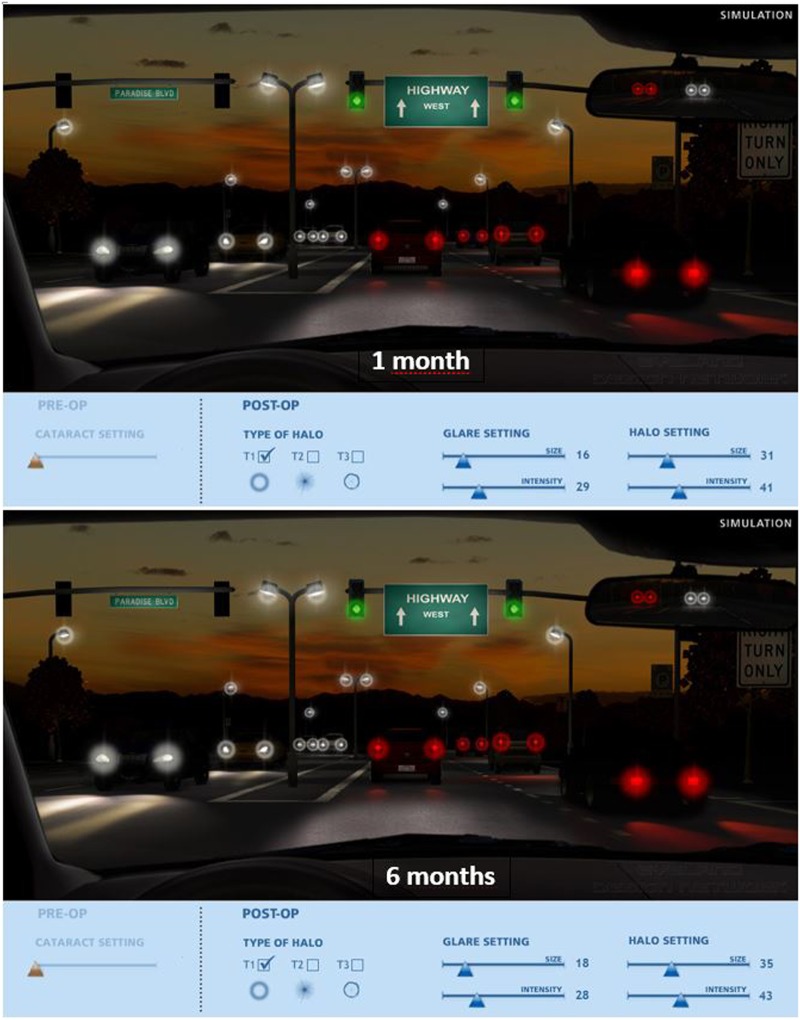
Average simulation of glare and halos at 1 and 6 months after surgery

At 3 and 12 months post-surgery, the majority of the patients rated their vision quality as good or very good at all distances. Halos were observed by 26.4% of the patients at the 1-month postop examination; however, this percentage dropped to 12.3% after 6 months. In terms of severity, 14.5% of patients reported severe symptoms after one month, but the figure dropped to 7.5% after three months. The dysphotopic subjective evaluation results from the questionnaire were relatively low and comparable with published data [17, 18]. The majority of patients reported that other types of dysphotopsia were mild or did not cause any disturbance. Distortion and multiple images were rare. Patients were tested subjectively to assess their night vision using a halo and glare software simulator, and asked to scale their observations. Halo size, type (type 1, 2 and 3), and intensity as well as glare size and intensity were scaled at 1-month and 6-month postop (Fig. 2). From a scale of 1 to 100, halo size ranged from 31 at 1 month to 35 at 6 months. 77% were small (Type 1) and remained unchanged at 6 months (76%). Halo intensity was scaled at 41 (1 month) and 43 at 6 months. Glare size was smaller (16 for 1 month and 18 at 6 months) and intensity was scaled at 29 at 1 month and 28 at 6 months. Overall, there were no statistically significant differences in the parameters evaluated between the 1-month and 6-month postoperative visits (*p* > 0.05).

### Patient satisfaction outcomes

**Fig. 3:**
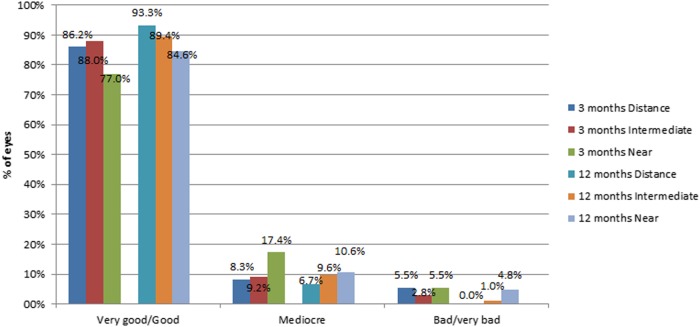
Distribution of the patient’s satisfaction outcomes at 3 and 12 months after surgery in the analysed sample

Postoperative patient satisfaction for visual outcomes at all distances was globally very high (Fig. 3). A large majority of patients were spectacle free for far (3 months: 89.9%, 12 months: 95.2%), intermediate (3 months: 94.5%, 12 months: 95.2%) and near visual tasks (3 months: 87.0%, 12 months: 83.7%). 12 months post-surgery, 90.4%, 95.2% and 73.1% of patients stated that they never had to wear glasses for far, intermediate and near visual activities, respectively.

### Vector analysis of astigmatic changes

The SIA was assessed using the Alpins method [14, 15]. Table 2 summarizes the results of vector analysis of astigmatic changes. Differences between both TIA and SIA were not statistically significant at all follow-up visits (*p* > 0.05). DV values were stable between 0.58 D (±0.28) and 0.61 D (±0.31). Mean postoperative CI ranged between 0.94 and 1.09, showing a slight trend to overcorrection which was concordant with the mean negative values of postoperative ME (ranging from −0.18 to −0.12 D). A statistically significant correlation was found between the magnitude of DV and postoperative manifest refractive cylinder (1 M: *r* = −0.79, *p* < 0.0001; 3 M: *r* = −0.85, *p* < 0.0001; 6 M: *r* = −0.82, *p* < 0.0001; 12 M: *r* = −0.82, *p* < 0.0001).

**Table 2:** Summary of the outcomes of the vector analysis of astigmatic changes occurring with surgery in the sample evaluated

Mean (SD)	1 month	3 months	6 months	12 months
SIA (D)	1.54 (0.90)	1.52 (0.93)	1.58 (0.89)	1.57 (0.93)
TIA (D)	1.55 (0.92)	1.55 (0.91)	1.53 (0.91)	1.55 (0.91)
DV (D)	0.58 (0.28)	0.61 (0.31)	0.61 (0.28)	0.59 (0.30)
ME (D)	−0.12 (0.48)	−0.18 (0.49)	−0.15 (0.47)	−0.16 (0.48)
AE (°)	−0.72 (14.77)	−0.57 (14.11)	−1.68 (12.76)	−2.95 (13.83)
CI	1.09 (0.84)	1.00 (0.63)	0.98 (0.40)	0.94 (0.34)
IoS	0.55 (0.90)	0.53 (0.66)	0.48 (0.44)	0.45 (0.29)
FE (D)	0.75 (0.81)	0.75 (0.82)	0.78 (0.79)	0.82 (0.89)
CA	1.22 (1.01)	1.28 (0.78)	1.23 (0.66)	1.25 (0.71)

*SD* standard deviation, *SIA* surgically induced astigmatism, *TIA* targeted induced astigmatism, *DV* difference vector, *ME* magnitude of error, *AE* angle of error, *CI* correction index, *IoS* index of success, *FE* flattening effect, *CA* coefficient of adjustment

### Complications

The IOL position, both tilt and decentration, was evaluated, by slit lamp examination, during the first postop visit and at M1, M3, M6 and M12 visits. Mean IOL decentration during the follow-up ranged from 0.6 mm ± 0.6 at 1 day postoperatively to 0.3 mm ± 0.3 at 6 months after surgery. Two patients had a tilted IOL at 1 day after surgery (one <5° and one >5°). 1 patient showed an IOL tilt of >5° at 6 and 12 months after surgery. 93.8% of IOLs implanted rotated ≤5° postoperatively. Three (3.7%) patients had an IOL successfully repositioned after rotation (5°, 15° and 38°).

Seventeen eyes (7.5%) in nine patients required Nd:YAG laser capsulotomy due to clinically detected levels of PCO. The time between cataract surgery and Nd:YAG ranged from 59–380 days. None of the adverse events reported during the trial were specifically related to the AT LISA tri toric IOL.

## Discussion

We evaluated the level of visual rehabilitation and patient satisfaction outcomes achieved with a diffractive trifocal toric IOL during a 12-month period in a large sample of eyes with significant amounts of corneal astigmatism. The data are consistent with those evaluating the same type of diffractive trifocal toric IOL at 3 months post-surgery [8–11]. Compared to other models of multifocal diffractive and refractive IOLs, the distance outcomes are similar or better than those reported by different authors [17–23]. In this study, when near vision was measured at 40 cm, mean postoperative binocular UNVA and DCNVA values were 0.16 logMAR and 0.15 logMAR, respectively. This outcome is consistent with monocular values obtained by Mojzis et al. [10] at 3 months after surgery (UNVA: 0.16 logMAR; CDVA: 0.15 logMAR) and binocular values obtained by Kretz et al. [11] (mean binocular UNVA: 0.10 logMAR at 3 months) and by Höhn et al. [12] (mean binocular UNVA: 0.09 logMAR at 12 months) in their series also evaluating the AT LISA trifocal toric IOL. Likewise, near visual outcomes were also consistent with those obtained with other models of bifocal and trifocal toric IOLs [17–23]. Excellent intermediate visual results were obtained in our study, with mean postoperative binocular UIVA of 0.06 logMAR and DCIVA values of 0.04 logMAR. Mojzis et al. [10] reported mean 3 month monocular UIVA of 0.08 logMAR and DCIVA of 0.07 logMAR, measured at 80 cm, and Kretz et al. [11] found mean binocular values of 0.08 and −0.03 logMAR also at 3 months postoperatively, but measured at 66 cm. Höhn et al. [12] obtained a mean UIVA of 0.00 logMAR at 12 months after the implantation of the AT LISA trifocal toric IOL. The data presented demonstrate the visual superiority at intermediate distances of the AT LISA tri toric 939MP IOL compared to bifocal toric IOLs [9, 17, 22]. Marques and colleagues [17] found, at 6 months post implantation of a diffractive bifocal IOL, a mean UIVA of 0.18 ± 0.09 logMAR and Shimoda et al. [22] reported a mean UIVA of 0.20 ± 0.09 at 70 cm at 3 months post implantation of another bifocal diffractive toric IOL.

The predictability of the refractive correction achieved was also good: 79.7% of eyes had a sphere and cylinder value within ±0.50 D at 12 months. This data is consistent with the results of previous studies evaluating the same and other types of multifocal IOLs [8–12, 17–24]. Mojzis et al. [16] found that 86.67% of eyes implanted with the non-toric AT LISA trifocal IOL had a postoperative spherical equivalent within ±0.50 D, whereas Law et al. [25] reported in another series a postoperative spherical equivalent ranging from −0.50 to +0.75 D at all postoperative visits of a 6-month follow-up in all eyes. A total of 78.6 and 92.9% of eyes implanted with an apodized +3 D addition toric diffractive IOL had a postoperative refractive cylinder of ≤0.50 and ≤1.00 D, respectively [26]. Bellucci et al. [27] confirmed that the refractive cylinder after implantation of the bifocal AT.LISA toric IOL was <1.00 D in 80.9% of eyes.

Vector analysis of astigmatic changes showed a slight trend to overcorrection: mean negative ME, mean CI of >1, and CA >1. It appears to be the main factor contributing to the slight residual postoperative cylinder (mean: −0.36 D) as a significant correlation was found between the residual cylinder and DV at all visits of the postoperative follow-up and no significant changes in corneal curvature were found at the end of follow-up. This slight trend to overcorrection has been also reported in previous studies evaluating the same trifocal toric IOL [10, 28]. Mojzis et al. [10] reported similar values for TIA (1.87 D ± 1.76) and SIA (1.92 D ± 1.55) to the values found in this report (TIA:1.54 D ± 0.91; SIA: 1.58D ± 0.89). Rotation stability of the trifocal toric IOL was good with 93.8% of the IOLs rotating ≤5° during the 12-month follow-up. This is consistent with the outcomes reported by Höhn et al. [12] that confirmed the good stability of the same trifocal toric IOL, with no patient showing an IOL rotation of >5° at 12 months.

Contrast sensitivity (CS) measurements are important to determine whether there is a possible loss of light transmission after IOL implantation and a subsequent impact on visual acuity. CS function, following implantation of the AT LISA tri toric 939MP is in the normal range. Visual acuities ≥0.22 logMAR were observed in the defocus range of AT LISA tri toric 939MP from −3.0 to +1.0 D; there was no visual acuity loss at any functional distance. The defocus curve shows a smooth transition from near to distant, similar to the defocus curves of trifocal non-toric IOLs [16, 25, 29–31], demonstrating that the addition of the toric component to the IOL design has no effect on visual acuity outcomes. A significant increase in the depth of focus allows excellent reading performance, comparable to diffractive and refractive bifocal and trifocal toric IOLs [17, 24, 29–32]. Dysphotopic phenomena are more common with multifocal IOLs than with monofocal IOLs. The design of diffractive multifocal IOLs, in particular the design of the ring zones, is important [17]. Can et al. [18] suggested that the design of the diffractive steps, with a soft transition, could explain the observed success in reducing visual symptoms found for certain diffractive multifocal IOLs. In our study patients were disturbed to a certain extent by halos, however, probably due to neural adaptation [25], they decreased over time.

Patient satisfaction is paramount, particularly in the case of trifocal toric IOLs because patients have high expectations and, in general, desire full spectacle independence. In this mutlicentre study, patients achieved excellent levels of spectacle independence, ranging between 73.1% of patients for near distance and 95.2% for intermediate distance at the 12-month postoperative visit.

In conclusion, the trifocal toric diffractive IOL AT LISA tri toric 939MP is an effective option for the restoration of the distance, intermediate and near visual function after cataract surgery in eyes with corneal astigmatism, providing high levels of quality of vision at all distances and high level of spectacle independence. Although the correction of astigmatism is very effective, a slight trend to overcorrection was observed. Improvements in effective lens position (ELP) calculations in future developments of the algorithms of power calculation of this IOL may help.

### Summary

#### What was known before:

- Bifocal Multifocal IOLs had significant percentage of patients complains for low quality of vision due to halos and glare and do not provide intermediate distance.
- Extended Depth of Focus IOLs are approved only for distance and intermediate vision.
- Toric IOLs correction was normally adopted for astigmatism correction over 2 diopters.

#### What this study adds:

- Trifocal Toric IOLs provide best refractive outcomes for far intermediate and near vision Toric correction should be applied when 0,75 diopters of corneal astigmatism is detected on corneal map Advance technology adoption provides better biometry outcomes to get emmetropia,or +/− 0,50 diopter, after cataract surgery.

## Electronic supplementary material

Figure 1 (supplemental)

Figure 1 (supplemental)
