# Supplementary figures and images for: Patient outcomes following implantation with a trifocal toric IOL: twelve-month prospective multicentre study
Source: Eye (Lond). 2018 Sep 6;33(1):144–53. doi: 10.1038/s41433-018-0076-5 (PMC6328597; doi:10.1038/s41433-018-0076-5)

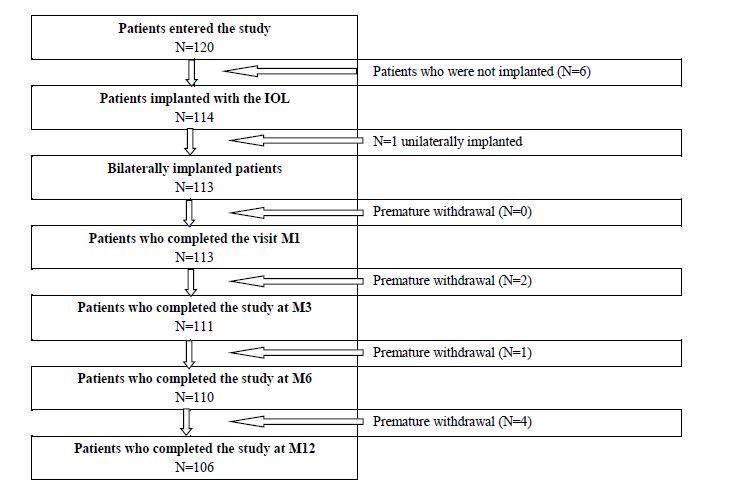

Supplement: Supplementary file 1 — Figure 1 (supplemental) [file 41433_2018_76_MOESM1_ESM.jpg]
