# Supplementary material for: Patient outcomes following implantation with a trifocal toric IOL: twelve-month prospective multicentre study
Source: Eye (Lond). 2018 Sep 6;33(1):144–53. doi: 10.1038/s41433-018-0076-5 (PMC6328597; doi:10.1038/s41433-018-0076-5)
Supplement: Supplementary file 2 — Figure 1 (supplemental) [file 41433_2018_76_MOESM2_ESM.docx]

Fig. 1 (supplemental data online) - Patient selection for the multicentre prospective study
